# Supplementary material for: DMRforPairs: identifying Differentially Methylated Regions between unique samples using array based methylation profiles
Source: BMC Bioinformatics. 2014 May 15;15:141. doi: 10.1186/1471-2105-15-141 (PMC4046028; doi:10.1186/1471-2105-15-141)
Supplement: Additional file 2 — DMRforPairs output for the comparison of A431-MCF7 and NA17018-NA17105. Please start from the HTML files in each folder. Available via the BMC Bioinformatics website. [file 1471-2105-15-141-S2.zip › 1394847754114233_MOESM2_ESM/A431_MCF7/figures/4487.pdf]

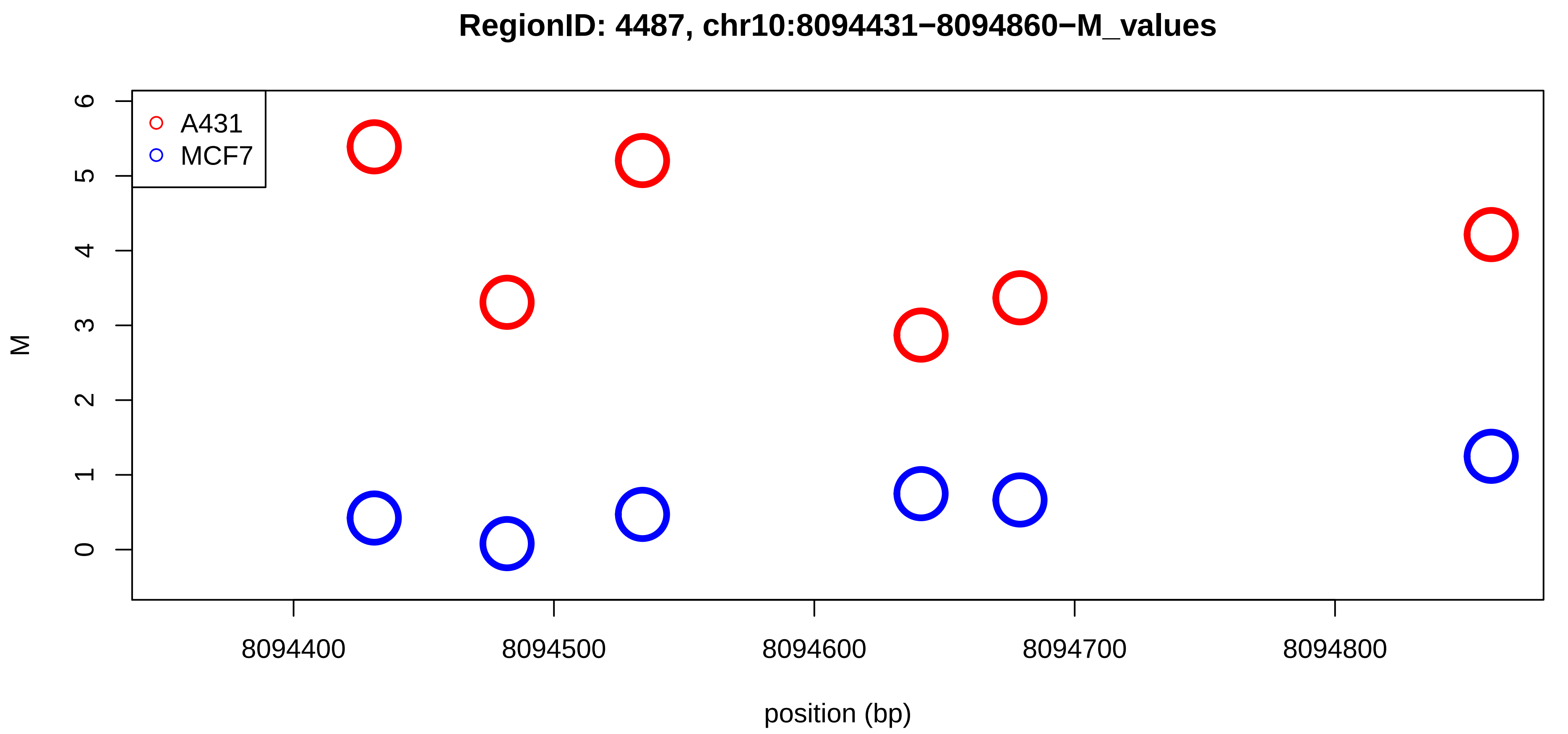
RegionID: 4487, chr10:8094431−8094860−M_values
A431
MCF7
M
0
1
2
3
4
5
6
8094400
8094500
8094600
8094700
8094800
position (bp)

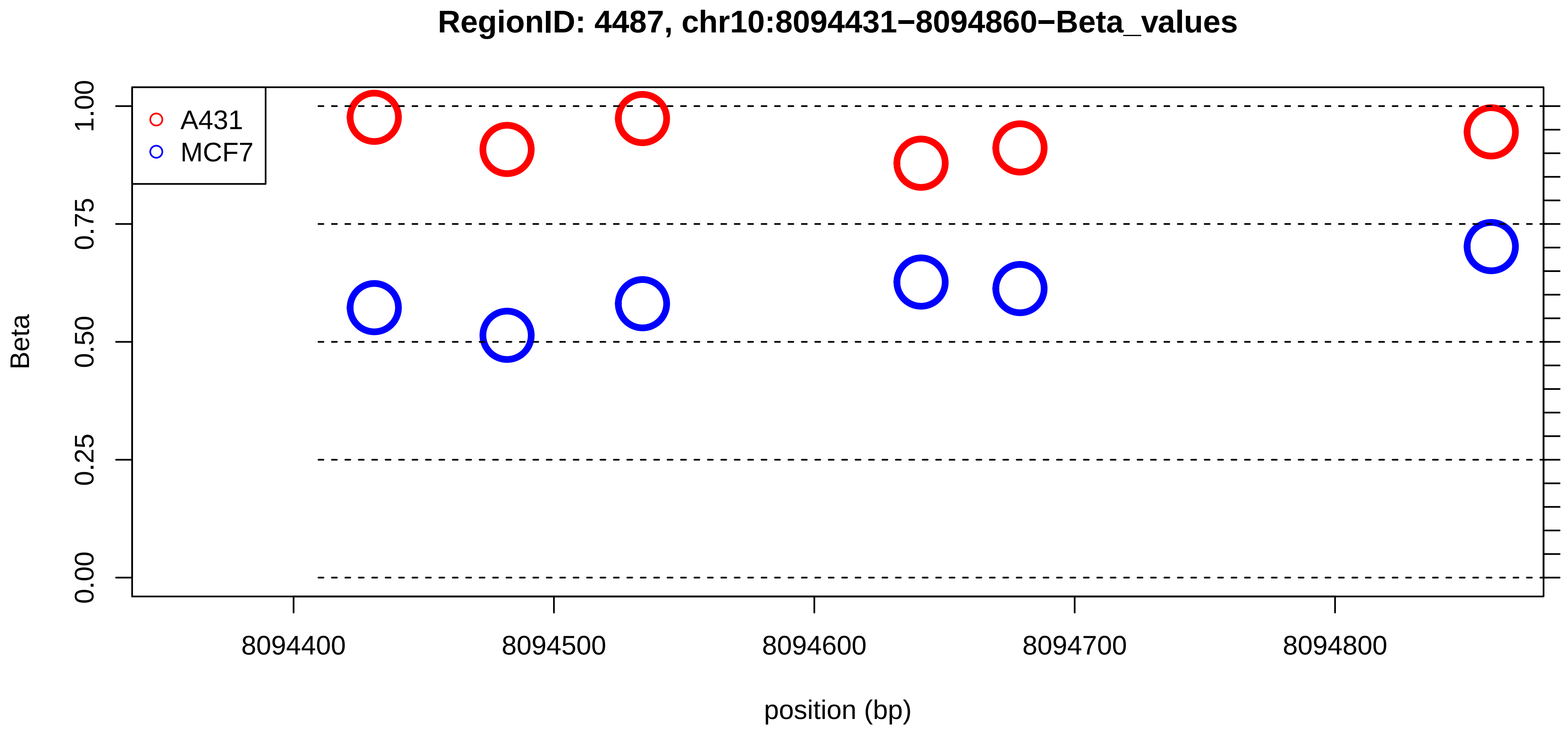
RegionID: 4487, chr10:8094431−8094860−Beta_values
A431
MCF7
Beta
0.00
0.25
0.50
0.75
1.00
8094400
8094500
8094600
8094700
8094800
position (bp)

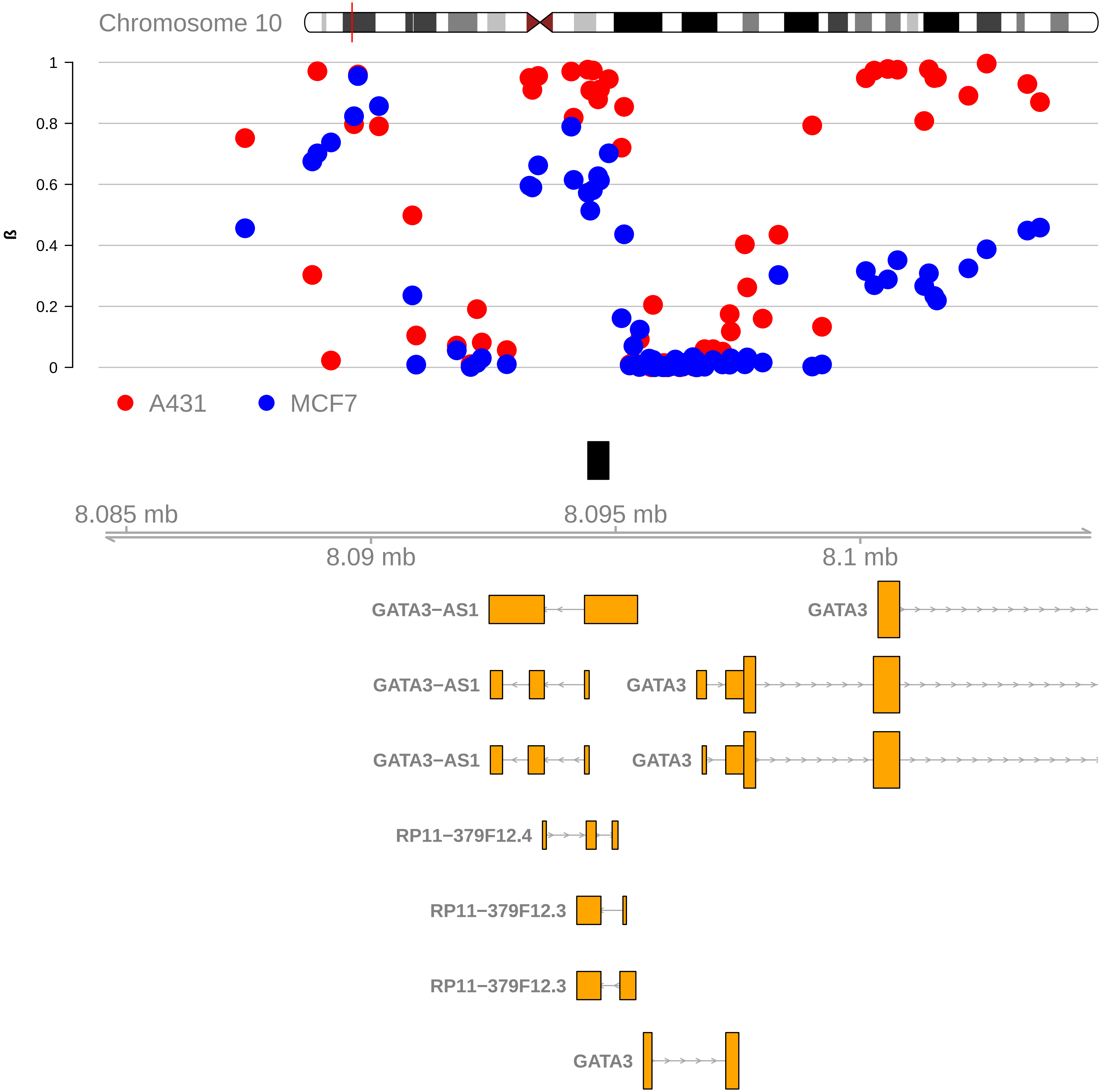
Chromosome 10
1
0.8
0.6
0.4
0.2
0
ß
A431
MCF7
8.085 mb
8.095 mb
8.09 mb
8.1 mb
GATA3−AS1
GATA3
GATA3−AS1
GATA3
GATA3−AS1
GATA3
RP11−379F12.4
RP11−379F12.3
RP11−379F12.3
GATA3
